# Patients’ Perspectives, Experiences, and Concerns With Perianal Fistulae: Insights From Online Targeted-Disease Forums

**DOI:** 10.1093/crocol/otad073

**Published:** 2023-11-15

**Authors:** Carine Khalil, Welmoed K van Deen, Taylor Dupuy, Gaurav Syal, Corey Arnold, Susan E Cazzetta, Pradeep P Nazarey, Christopher V Almario, Brennan M R Spiegel

**Affiliations:** Division of Health Services Research, Cedars-Sinai Medical Center, Center for Outcomes Research and Education (CS-CORE), Los Angeles, CA, USA; Division of Health Services Research, Cedars-Sinai Medical Center, Center for Outcomes Research and Education (CS-CORE), Los Angeles, CA, USA; Erasmus School of Health Policy & Management, Rotterdam, The Netherlands; Division of Health Services Research, Cedars-Sinai Medical Center, Center for Outcomes Research and Education (CS-CORE), Los Angeles, CA, USA; Cedars-Sinai Medical Center, Department of Medicine, Los Angeles, CA, USA; Medical Imaging Informatics, Department of Radiology, UCLA, Los Angeles, CA, USA; Takeda Pharmaceuticals USA, Inc., Lexington, MA, USA; Takeda Pharmaceuticals USA, Inc., Lexington, MA, USA; Division of Health Services Research, Cedars-Sinai Medical Center, Center for Outcomes Research and Education (CS-CORE), Los Angeles, CA, USA; Division of Health Services Research, Cedars-Sinai Medical Center, Center for Outcomes Research and Education (CS-CORE), Los Angeles, CA, USA

**Keywords:** perianal fistula, social media, netnography, patient perspectives, Crohn’s disease

## Abstract

**Background:**

Perianal fistulae can undermine physical, emotional, and social well-being in patients with Crohn’s disease and are challenging to manage. Social media offers a rich opportunity to gain an in-depth understanding of the impact of perianal fistulae on patients’ daily lives outside of controlled environments. In this study, we conducted social media analytics to examine patients’ experiences with perianal fistulae and assessed the impact of perianal fistulae on patients’ behavior and overall well-being.

**Methods:**

We used a mixed-method approach to examine 119 986 publicly available posts collected from 10 Crohn’s disease forums in the United States between January 01, 2010 and January 01, 2020. Discussions related to Crohn’s perianal fistulae were retrieved. We randomly selected 700 posts and qualitatively analyzed them using an inductive thematic approach. We then applied a latent Dirichlet allocation probabilistic topic model to explore themes in an unsupervised manner on the collection of 119 986 posts.

**Results:**

In the qualitative analysis, 5 major themes were identified: (1) burden of perianal fistula; (2) challenges associated with treatment; (3) online information seeking and sharing; (4) patient experiences with treatments; and (5) patients’ apprehension about treatments. In the quantitative analysis, the percentages of posts related to the major themes were (1) 20%, (2) 29%, (3) 66%, and (4) 28%, while the topic model did not identify theme 5.

**Conclusions:**

Social media reveals a dynamic range of themes governing patients’ perspectives and experiences with Crohn’s perianal fistulae. In addition to the biopsychosocial burden, patients frequently express dissatisfaction with current treatments and often struggle to navigate among available management options.

## Introduction

Crohn’s disease (CD) is a chronic inflammatory disease of the intestine characterized by transmural inflammation that can disrupt the mucosal integrity of the intestine, leading to complications such as abscesses and perianal fistulae.^1^ The prevalence of Crohn’s perianal fistulae (CPF) is estimated at 11%–21% within 1 year of CD diagnosis and between 21% and 54% for patients within 20 years of the initial CD diagnosis.^2,3^ The presence of CPF is associated with higher health care costs and resource use.^4–7^ Although perianal fistulae are common in CD, their management remains challenging. Patients often have concerns regarding perianal fistulas and the associated treatment options.^8^ Currently available medical and surgical treatments have limited effectiveness and recurrence tends to occur in 50% of the cases.^9^ The symptoms associated with perianal fistula, including pain, perianal itching, bleeding, and discharge of pus, significantly impair patients’ quality of life.^10,11^ Additionally, patients with CPF often deal with anxiety and other psychological disorders.^12^

Social media can be a valuable source of information to learn about patients’ experiences as patients frequently discuss their symptoms and concerns online, sometimes anonymously and without fear of embarrassment or shame. Social media platforms offer participants a virtually borderless environment where geographically diverse members can meet, share information, and communicate freely. It has grown commonplace for people with chronic conditions to use social media to share their personal experiences and to seek advice and encouragement from others with similar health issues and concerns.^13–15^ The role of social media in health care has expanded over the past decade and is now used for patient clinical trial recruitment, measurement of consumer sentiment, patient education, patient monitoring, management of patient care, and epidemiological research.^16–18^ Despite the expanse of publicly available information from social media platforms and the increased use of social media in the inflammatory bowel disease (IBD) community,^19^ there has been limited research to analyze social media as a “big data” resource to understand the patient experience in CD and more specifically in CPF.

In this study, we sought to learn about patients’ perspectives, concerns, and experiences with CPF and their existing treatments. We conducted a social media netnography to gain an understanding of how CPF affects patients’ quality of life and overall well-being and how patients with CPF navigate their daily challenges. Social media netnography is a research methodology that adapts ethnographic principles and methods for the study of free-range, nonexperimental communities created through computer-mediated social interaction.^20^

## Methods

### Data Collection

We used a systematic approach to identify targeted forums that generate discussions around CPF on the Google search engine. We used key terms such as “Peri-anal fistula forum,” “Crohn’s disease fistula forum,” “Crohn’s Perianal Fistula,” “Anal fissure forum,” “Perianal fissure forums,” and “Fistulizing forums” to search for relevant forums. For every Google search, we reviewed the first 30 results based on Click Through Rates.^21^ The content of each identified forum was evaluated based on our inclusion and exclusion criteria. We included health-related forums that discussed CPF, were hosted in the United States, and were public-facing without requiring login information. We excluded forums with low activity such as individual blogs that do not promote user interactions, nonhealth-related forums, and forums that generate discussions for nonhumans (eg, perianal fistulae in dogs). In total, 10 forums were included in our study (Supplementary Appendix 1). Relevant threads and subthreads were identified within each forum by searching for the keywords described earlier. We collaborated with Mozenda (www.mozenda.com), a social media data analysis service, to extract posts from the identified forums. Only US posts from the last 10 years were selected from each forum and compiled in a data set. In total, 119 986 publicly available posts were collected from 10 CD forums between January 01, 2010 and January 01, 2020. No identifying information such as names and addresses were extracted and included in the data set. Table 1 gives an overview of the data extraction strategy.

**Table 1.** Data extraction strategy.

Keywords	“Peri-Anal fistula forum”	“Crohn’s disease fistula forum”	“Anal fissure forum”	“Crohn’s disease forum”
Results reviewed	30 results for each keyword search (3 pages of 10 results each)			
Inclusion criteria	User-generated discussions Relevant forum discussion			
Exclusion criteria	Outside the United States Nonhuman-related sites			
Identified and reviewed forums	26 excluded	28 excluded	28 excluded	29 excluded
Relevant forums included in the study	4 included	2 included	2 included	2 included

### Data Analysis

#### Qualitative methods

We used an open coding technique to analyze patient discussions regarding perianal fistulae. A total of 700 posts were randomly selected from the 119 986 available posts and analyzed using an inductive thematic analysis. The analyses were performed by an experienced social scientist (C.K.) with formal training in qualitative methods. The qualitative data were iteratively reviewed to achieve total immersion in the discussions. Throughout the reading, words and sentences were manually highlighted and coded. Key labels were inductively identified and applied within the unstructured data. After sorting, combining, and refining the generated codes, a set of inductive themes and subthemes were defined and justified with individual posts.^22^ Themes and subthemes were iteratively revised and refined throughout the analysis process. Data saturation was achieved after analyzing 400 posts. This was confirmed after analyzing 300 additional randomly selected posts. To ensure validity, findings were discussed during several team debriefing sessions, in the presence of 5 coauthors (C.K., W.D., T.D., G.S., and B.S.). Data summaries were also presented to the other coauthors (S.C. and P.N.) in order to share perspectives on the insights obtained. Table 2 shows examples from the coding process.

**Table 2.** Examples from the coding process.

Posts and coding process	Subthemes	Themes
*I had* ***an attempt at repair*** *a couple of years ago that* ***was unsuccessful*** *. Mine was very low and there was so much scar tissue it didn’t work*	Unsuccessful surgical procedure	Challenges associated with treatments
*To quote the surgeon I consulted* “***If I cut it out, I’d just create 10 more***”	Unsuccessful surgical procedure	
*I’ve had a* ***recurring fistula*** *since September 2009*	Recurring fistula	Challenges associated with treatments
*I have a* ***recurring perianal abscess*** *with 3 fistula tracts*	Recurring fistula	
*I* ***hope you have found remission …*** *good feeling knowing* ***we are not alone***	Peer support	Seeking/sharing online information
*I* ***learned*** *so much* ***from your lives and kindness***	Peer support	
“*are there****any updates on your Stem Cell Treatment****?!?*”	Novel therapies	Seeking/sharing online information
“*I was hoping to****hear about advancement flap surgeries***”	Treatments	

#### Quantitative methods

In addition to the qualitative analysis, we used a latent Dirichlet allocation (LDA) topic model to explore themes within the collection of the 119 986 publicly available posts in an unsupervised manner. In the LDA framework, words that commonly co-occur in the same context are grouped into “topics,” which are defined as multinomial distributions over words.^23^ In our work, we determined that a model with 50 topics best fitted the data. These topics were manually examined by an expert panel of medical professionals and researchers. We reviewed the top 20 words within a topic (ranked by their probability), which dominate a topic’s semantic meaning. To further understand a topic’s semantic meaning in the context of the collected posts we reviewed the 10 posts in which the topic had the highest prevalence. Topics that were uninformative or generically related to CD only rather than CPF specifically were not used for further analysis. The remaining topics were matched to one or more of the themes identified in the qualitative analysis. Table 3 shows examples from the topic modeling process.

**Table 3.** Examples from the topic modeling process.

Topics	Themes
Insurance, company, health, disability, medical, month, year, know, coverage, need, covered, assistance, expensive, approved, help, drug, time, program, pharmacy, companies	Challenges ­associated with treatment
Stomach, feel, symptoms, nausea, having, time, fever, started, today, pains, hours, really, normal, eat, diarrhea, week, worse, got, vomiting, eating	Burden of ­perianal fistula
Sorry, hugs, soon, hear, know, things, feel, think, hopefully, time, sounds, news, help, really, sure, great, fingers, happy, thoughts, prayers	Seeking/sharing online ­information

#### Ethical considerations

##### Institutional review board approval and ethical considerations

This study was reviewed by the Cedars-Sinai Medical Center Institutional Review Board (STUDY00000968) and it was deemed exempt from review, as it did not meet the definition of “human subject research” under the Department of Health and Human Services or U.S. Food and Drug Administration regulations.

## Results

### Quantitative Analysis

Of the 50 topics learned by the model, 6 were uninformative and 27 were related to general CD topics rather than discussions about perianal fistulae specifically. In total 17 topics were deemed relevant to be included for further analysis, each of which was matched to one or more of the themes identified in the qualitative thematic analysis. Of those, 14 were related to online information seeking and sharing; 7 were related to patients’ experiences with treatments; 5 were related to challenges associated with treatment; and 3 were related to the burden of perianal fistulae. Overall, we found that 66% of posts were related to online information seeking and sharing; 29% of posts were related to challenges associated with treatment; 28% described patients’ experiences with treatments; 20% were related to the burden of perianal fistulae; and 25% could not be classified (Table 4).

**Table 4.** Frequency of themes based on LDA topic model results. Individual posts were deemed to mention a topic if more than 15% of the words in the post were attributed to the topic.

Themes	*n* of posts	% of posts
No theme assigned	26 485	25
At least 1 theme assigned	79 772	75
Burden of perianal fistula	21 294	20
Challenges associated with treatment	30 746	29
Online information seeking and sharing	69 792	66
Patient experiences with treatments	29 666	28
More than 1 theme assigned	48 396	46
Total	106 257	100

### Qualitative Analysis

**Figure 1.**
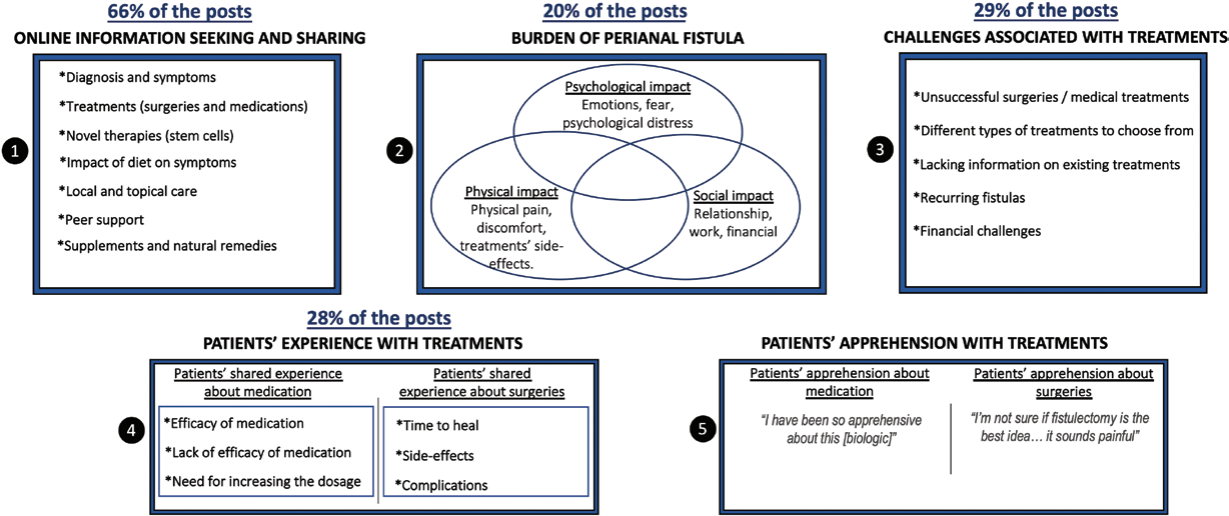
Themes identified in the qualitative analysis. We didn’t include the percentage of “Patients’ apprehension with treatments” as this theme was only generated by the qualitative analysis.

In our qualitative analysis, 5 major themes were identified related to perianal fistulae (Figure 1): (1) burden of perianal fistula; (2) challenges associated with treatment; (3) online information seeking and sharing; (4) patient experiences with treatments; and (5) patients’ apprehension about treatments. (We did not include the percentage of “Patients’ apprehension with treatments” as this theme was only generated by the qualitative analysis.)

#### The burden of perianal fistula

The chronic aspect of perianal fistulae and the associated symptoms caused a wide range of physical, psychological, and social concerns among patients. These include fear and psychological distress related to the disease and treatments (“*There were day of constant tears . . . why me?*”; “*sorry for my depression outburst*”; “*I feel scared and nervous about the thought of having a fistulotomy*”), physical pain and discomfort (“*I had terrible pain*”; “*the seton is very very painful*”; “*standing and sitting are pretty unbearable*”; “*there is no relief*”; “*I dealt with drainage for years*”), embarrassment and social avoidance (“*I am not interested in finding a partner as I would not let them go near me*”), as well as concerns regarding work absenteeism due to sickness (“*I can’t take many days off work*”).

#### Challenges associated with treatments

In addition to the biopsychosocial impact of the perianal fistulae, patients with perianal fistulae face many challenges related to their treatment. They discussed the low success rate of medical and surgical treatments (“*I have over 10 surgeries of draining and adding glue*”), high fistula recurrence rates (“*My fistulas are now recurring, as soon as one heals up, I get two more*”), and financial challenges associated with treatments (“*I am going to have to pay $1600 and being off work for a while is going to be hard financially for us*”). Patients also express a lack of adequate information regarding perianal fistulae treatment options (“*If I knew prior to my surgery what I know now I would have opted to go the medication route first as well*”) and increasing uncertainty about how to make an informed treatment decision (“*I try to gather as much information as I can about it. Likewise with the glue etc. also there has been discussion on the advancement flap*”).

#### Online information seeking and sharing

Given the lack of adequate information regarding perianal fistulae treatments, patients find themselves seeking and sharing online information about their disease. They increasingly use targeted-disease forums to share and seek information regarding perianal fistulae diagnosis and symptoms (“*I don’t understand why I’m still leaking so badly in the front*”), conventional treatments such as surgeries and medications (“*I was hoping to hear about advancement flap surgeries*”), novel therapies (“*are there any updates on your Stem Cell Treatment?!?*”), impact of diet on perianal fistulae symptoms (“*you have to stay on high-fiber diet*”), supplements and natural remedies (“*my most effective supplements are probiotics, a soluble fiber such as psyllium, fish oil, and vitamin D*”; “*sitz baths with lots of Epsom salts as often in a day that you can*.”), as well as local and topical care to ease their symptoms (“*I did respond to the topicals I was given … it helps irritation*”). Patients also use these forums to seek support from peers who are in similar situations. This can sometimes alleviate their concerns and worries (“*any and all encouragement and suggestions are appreciated*”).

#### Patients’ experience with treatments

Additionally, patients use online forums to share their experiences with perianal fistulae treatments, potentially influencing other patients’ treatment decisions and behaviors. While some patients highlight the efficacy of medications (“*mine were healed when I went on this [biologic]*”), others express their dissatisfaction with medications due to their lack of efficacy (“*I take prednisone to reduce symptoms, but it doesn’t really help*”; “*this [biologic] isn’t working as they said it would*”), and the need for increasing their dosage and/or frequency (“*before the weekly dosage, the fistula would open again*”). Also, many patients reported their negative experiences with surgeries. They talked about the repeated abscesses “*I have a new abscess deep in my buttock*,” and the need for multiple surgeries when the fistula is complex “*there is a lot of uncertainty surrounding fistulas, if complex [fistula], then repeated surgeries are likely*” and the accompanying burden “*After 8 surgeries the pain in the left never went away.*” In addition to the associated complications “*unbelievable pain after each bowel movement*” and the requisite maintenance “*I hate ‘seton’ for sure because it’s high maintenance*,” some patients commented on a prolonged recovery time after surgical procedures (“*it’s been a year and it feels like I’m only at the beginning of recovery*”).

#### Patients’ apprehension with treatments

Patients’ shared dissatisfaction with treatments on social media might increase other patients’ concerns and worries “*read that they stick a probe through the fistula to perform the surgery - it sounds brutal and barbaric.*” Many posts highlighted patients’ apprehension about existing treatments. Although some appear to be hesitant about starting medication (“*I am a little apprehensive, few people are on this drug or know about it*”; “*I had been so apprehensive about this [biologic]*”), others seem concerned about surgeries “*whenever a family member faces surgery it can be a very apprehensive time*”; “*I’m not sure if fistulectomy is the best idea … it sounds painful*”). Such concerns often lead to eliciting medical advice from peers who share their “information” and/or experience with these treatments.

## Discussion

Social media netnography reveals a wide range of themes related to patients’ perspectives and experiences with CPF. After collecting 119 986 posts from 10 targeted-disease forums, we identified and sorted common themes among related posts, qualitatively generated themes from a sample of 700 posts using an inductive thematic approach, and quantitatively examined the prevalence of each theme. We leveraged qualitative and quantitative analyses to explore and understand in depth a large repository of social media posts.

The use of a mixed-methods approach provides complementary results. The qualitative analysis offers a detailed understanding of how patients experience perianal fistulae, including its impact on the physical, psychological, and social levels, and how they perceive associated treatments as well as alternative remedies to alleviate their symptoms. The quantitative analysis enumerates the frequency of the different themes categorized within the data. One theme (patients’ apprehension with treatments) was identified in the qualitative analysis and not in the quantitative analysis, which highlights the relevance of the manual thematic analysis.

Consistent with prior work, this study also shows the importance of social media use in the IBD community.^19,24,25^ Patients use these platforms to talk about their concerns and challenges, share and seek medical information, and support each other. The concerns and challenges mentioned by patients throughout the years did not really change. Hence, by understanding patients’ experience with perianal fistulae, it becomes easier to identify actionable insights that can address patients’ needs in terms of educational material and improve their quality of life. For example, our data revealed the need to develop and disseminate information on how to cope with CPF, and how to navigate treatment options. Disseminating reliable information from trusted sources is crucial in a context where patients are often unsure of the quality of information posted online.^24^

Topic modeling reveals that patients predominantly use targeted-disease forums to seek and share information about CPF diagnosis and symptoms, medical treatments, diet, supplements and natural remedies, as well as topical care. They also use these forums to seek and share peer support. The analysis also shows that discussions around the burden of CPF, challenges associated with treatments, and experiences with treatments are prevalent. The burden of CPF and patients’ lack of adequate information regarding their diagnosis, symptoms, and treatment options highlight the need for developing educational material that can aid patients in treatment decision-making and help them cope with the biopsychosocial burden of perianal fistulae.

While many of our findings are consistent with what is known about CPF, most prior studies examining the impact of CPF on QOL were based on small sample sizes and done in research settings which is subject to Hawthorne bias.^26–28^ In our study, we examined nearly 120 000 posts made by people in free-range, nonexperimental settings, thereby allowing us to obtain a real-world assessment of the marked biopsychosocial impact of CPF. Moreover, we also examined the large corpus of posts using a LDA topic model which enabled us to quantify how often certain themes appeared online. This may inform investigators and patient advocacy groups when developing educational materials that are focused on the most prevalent themes that people with CPF talk about online.

Our study has limitations. First, by using social media platforms as a data source, we cannot confirm the diagnosis of individuals with CPF who posted on the identified platforms. However, high validity of self-reported diagnoses of chronic diseases was found in prior studies.^29–31^ Second, the study can only be generalized to individuals with CPF who also use social media. However, according to Pew Research Center, approximately 84% of individuals under the age of 30 and 81% under the age of 49 use social media.^32^ This study reveals the benefits of social media research, particularly in rare and chronic conditions. It helps to obtain insights from a large number of individuals with CPF across the United States. The anonymity offered by social media enables people who are more reserved to share their experiences and perspectives about specific topics related to CPF openly. However, by only focusing on forums that require login information, we might have under-captured user discussions that are generated on platforms that require identification.

In summary, social media netnography reveals a dynamic range of themes governing patients’ experience with CPF. This study helps identify unmet needs related to patient education related to CPF, particularly related to navigating the complexity of the available medical and surgical treatment options and coping with the biopsychosocial impact of CPF. Our findings may help clinicians and researchers develop targeted educational materials and support strategies that address these needs and improve the quality of life of patients suffering from this rare condition with significant burden. Our findings also highlight a need for further research to develop novel therapies for CPF.

## Supplementary Material

otad073_suppl_Supplementary_Appendix

## Data Availability

Data available in Supplementary Data.
