# Supplementary material for: Patients’ Perspectives, Experiences, and Concerns With Perianal Fistulae: Insights From Online Targeted-Disease Forums
Source: Crohns Colitis 360. 2023 Nov 15;5(4):otad073. doi: 10.1093/crocol/otad073 (PMC10693318; doi:10.1093/crocol/otad073)
Supplement: otad073_suppl_Supplementary_Appendix [file otad073_suppl_supplementary_appendix.docx]

**Appendix 1**

**List of selected forums**

1. <https://inflammatoryboweldisease.net>
2. <https://patient.info/forums/>
3. <https://www.crohnscolitiscommunity.org/>
4. <https://crohnsforum.com/>
5. <http://www.ibdsupport.org/forums>
6. <https://healthunlocked.com/tag/anal-fissure>
7. <https://anal-fissure.org/>
8. <https://www.healingwell.com/community/>
9. <https://www.reddit.com/r/CrohnsDisease/>
10. <https://www.reddit.com/r/IBD/>
